# The importance of genotype-phenotype correlation in the clinical management of Marfan syndrome

**DOI:** 10.1186/s13023-017-0754-6

**Published:** 2018-01-22

**Authors:** Víctor Manuel Becerra-Muñoz, Juan José Gómez-Doblas, Carlos Porras-Martín, Miguel Such-Martínez, María Generosa Crespo-Leiro, Roberto Barriales-Villa, Eduardo de Teresa-Galván, Manuel Jiménez-Navarro, Fernando Cabrera-Bueno

**Affiliations:** 1 Unidad de Gestión Clínica del Corazón, Hospital Universitario Virgen de la Victoria, Instituto de Investigación Biomédica de Málaga (IBIMA), Universidad de Málaga (UMA), CIBERCV Enfermedades Cardiovasculares, Málaga, Spain; 2 Unidad de Insuficiencia Cardiaca Avanzada y Trasplante Cardiaco. Servicio de Cardiología. CIBERCV. Instituto de Investigación Biomédica de A Coruña (INIBIC), Complexo Hospitalario Universitario de A Coruña (CHUAC), SERGAS. Universidade da Coruña (UDC). As Xubias, 15006 A Coruña, Spain

**Keywords:** Marfan syndrome, FBN-1, Hereditary aortopathy, Genetic testing, Ascending aortic aneurysm, Type a dissection

## Abstract

**Background:**

Marfan syndrome (MFS) is a disorder of autosomal dominant inheritance, in which aortic root dilation is the main cause of morbidity and mortality. Fibrillin-1 (FBN-1) gene mutations are found in more than 90% of MFS cases. The aim of our study was to summarise variants in FBN-1 and establish the genotype-phenotype correlation, with particular interest in the onset of aortic events, in a broad population of patients with an initial clinical suspicion of MFS.

**Material and methods:**

This single centre prospective cohort study included all patients presenting variants in the FBN-1 gene who visited a Hereditary Aortopathy clinic between September 2010 and October 2016.

**Results:**

The study included 90 patients with FBN-1 variants corresponding to 58 non-interrelated families. Of the 57 FBN-1 variants found, 25 (43.9%) had previously been described, 23 of which had been identified as associated with MFS, while the the remainder are described for the first time. For 84 patients (93.3%), it was possible to give a definite diagnosis of Marfan syndrome in accordance with Ghent criteria. 44 of them had missense mutations, 6 of whom had suffered an aortic event (with either prophylactic surgery for aneurysm or dissection), whereas 20 of the 35 patients with *truncating* mutations had suffered an event (13.6% vs. 57.1%, *p* < 0.001). These events tended to occur at earlier ages in patients with *truncating* compared to those with *missense* mutations, although not significantly (41.33 ± 3.77 vs. 37.5 ± 9.62 years, *p* = 0.162).

**Conclusions:**

Patients with MFS and *truncating* variants in FBN-1 presented a higher proportion of aortic events, compared to a more benign course in patients with *missense* mutations. Genetic findings could, therefore, have importance not only in the diagnosis, but also in risk stratification and clinical management of patients with suspected MFS.

**Electronic supplementary material:**

The online version of this article (10.1186/s13023-017-0754-6) contains supplementary material, which is available to authorized users.

## Background

In recent years, genetics has become the cornerstone for the screening and correct management of a good number of cardiovascular diseases [1]. Although genetics has been employed to a lesser extent in our speciality than in other areas, such as cardiomyopathy, its use in cases of hereditary or familial aortopathy has been clearly demonstrated [2].

Marfan syndrome (MFS) is the best known example of a hereditary syndromic aortopathy [3], and is the one most frequently encountered in daily practice, with a reported prevalence of 6.5/100,000 [4]. It is an autosomal dominant inheritance pattern disorder in which aortic root dilation, a characteristic major criterion and the main cause of morbidity and mortality, can lead to thoracic aorta aneurysm (TA) or Type A aortic dissection (TAAD). Other important signs of MFS include ectopia lentis (also a major criterion) and a series of systemic findings such as myopia, mitral valve prolapse, dural ectasia and skeletal abnormalities, all of which have degrees of involvement that may vary [5, 6]. It is known that a definitive diagnosis cannot always be reached solely based on clinical criteria, due the syndrome’s variable expression, its slow progression, or to screening undertaken at an early age when phenotype has not yet had time to manifest [7]. For these reasons a good knowledge of the genetic substrate could play a determinant role in early diagnosis.

The first mutation of FBN-1 associated with MFS [8, 9] was identified in 1991; this gene codifies fibrillin-1, a structural macromolecule that polymerizes to form microfibrils and contributes to the integrity and function of connective tissues [10]. Mutations in FBN-1 are found in more than 90% of MFS cases [11], which is why the Ghent criteria [6, 12] for MFS have added findings of a pathogenic variation in this gene to family history and the main clinical signs in order to establish a diagnosis. Moreover, it has recently been suggested that the type of FBN-1 variation could even condition the prognosis of these patients [13, 14]. Thus, a deeper knowledge of genetics and phenotypic expression could also facilitate identification of what are, from a cardiovascular perspective, more aggressive forms of the disorder.

The objective of our study was, therefore, to summarise the different FBN-1 variants and establish a genotype-phenotype correlation in a broad population of patients with MFS, with particular focus on the onset of aortic events.

## Methods

### Study population

This single-centre prospective cohort study included all patients studied in the Hereditary Aortopathy clinic of a tertiary referral hospital who had an initially suspected MFS and presented FBN-1 gene variants between September 2010 and October 2016.

The 532 patients who were studied at the clinic came from 248 non-interrelated families. Patients either presented with aortic complaints that developed at an early age in the absence of known risk factors and/or had family history of aortic events or sudden death, or were a first degree relative of a patient who presented these characteristics.

In the first visit, a thorough physical examination was performed in search of the clinical signs generally associated with syndromic aortopathies, and a transthoracic echocardiogram was also carried out. After this first approach, if the suspicion of hereditary aortopathy (syndromic or not) remained, a blood sample was taken for genetic testing. This was done for 294 patients, in 108 cases directed by clinical suspicion of MFS. All of participants were duly informed about the benefits and risks of having the test, and signed informed consent.

Definite diagnosis of Marfan syndrome was made in accordance with modified Ghent criteria, which state that in the presence of family history, the following findings lead to a definitive diagnosis of MFS: 1) ectopia lentis, or 2) aortic root dilation (Z-score ≥ 2 above 20 years, ≥ 3 below 20 years), or 3) a systemic score ≥ 7 (Table 1). In the absence of a family history, a combination of the following would be necessary: 1) aortic root dilation and ectopia lentis, 2) aortic root dilation and pathogenic FBN-1 mutation, 3) aortic root dilation and systemic score ≥ 7, or 4) ectopia lentis and causal FBN-1 mutation (in this case, as the relationship between the mutation and MFS cannot be established, it is defined as “ectopia lentis syndrome”) [12].

**Table 1:** Scoring of systemic features [12]

Wrist AND thumb sign/wrist OR thumb sign	3 points/1 point
Pectus carinatum deformity/Pectus excavatum or chest asymmetry	2 points/1 point
Hindfoot deformity/Plain pes planus	2 points/1 point
Pneumothorax	2 points
Dural ectasia	2 points
Protrusio acetabuli	2 points
Reduced US/LS AND increased arm/height AND no severe scoliosis	1 point
Scoliosis or thoracolumbar kyphosis	1 point
Reduced elbow extension	1 point
Facial features (3/5): dolichocephaly, enophthalmos, downslanting palpebral fissures, malar hypoplasia, retrognathia	1 point
Skin striae	1 point
Myopia >3 dioptres	1 point
Mitral valve prolapse (all types)	1 point
Maximum total: 20 points; score ≥ 7 indicates systemic involvement	

*US/LS* upper segment/lower segment ratio

Given the recognised variable clinical presentation of MFS depending on age, with lower expression in younger patients [7, 15], an analysis of baseline characteristics was undertaken for the whole population, above 18 years and below 18 years.

### Echocardiographic study

All patients had a transthoracic echocardiogram at each visit, using a GE Vivid T7 (General Electric Company, Connecticut, USA). These studies included M-mode, two dimensional (2D), spectral and color Doppler. In all cases, 2D measurements were taken at the level of the aortic annulus, sinuses of Valsalva, sinotubular junction and ascending aorta, in accordance with the recommendations of current guidelines [16, 17]. *Mild aortic dilatation* (MAD) was defined as an aortic root or ascending aorta of dimensions higher than the upper limit of the confidence interval at 95% of the distribution of a reference population (Z-score ≥ 2 [18, 19]), but without reaching surgical parameters as defined in current clinical practice guidelines, which is, in any case, 50 mm [17]. Thoracic aorta aneurysm (TA) was defined when the dimensions were higher. The existence of *mitral valve prolapse* (MVP) was assessed in all patients. The grade of valve regurgitation was assessed and classified as mild, moderate or severe, following American Society of Echocardiography grading [20].

### Genetic analysis

Once the blood samples for genetic analysis had been taken, they were studied in a single reference laboratory, using a massive parallel sequencing library that included 30 genes associated with aneurysm and aortic dissection, of which FBN-1 is one. The FBN-1 variants identified were confirmed by Sanger sequencing in both directions.

The variants were classified as *pathogenic* if they met the *criteria for causal FBN-1 mutations* in accordance with modified Ghent criteria (Table 2) [12]. The variants found have been reported following the nomenclature established in the American College of Medical Genetics (ACMG) recommendations [21, 22].

**Table 2:** Criteria for causal FBN1 mutation [11]

Mutation previously shown to segregate in Marfan family
De novo (with proven paternity and absence of disease in parents) mutation (one of the five following categories)
Nonsense mutation
Inframe and out of frame deletion/insertion
Splice site mutations affecting canonical splice sequence or shown to alter splicing on mRNA/cDNA level
Missense affecting/creating cysteine residues
Missense affecting conserved residues of the EGF consensus sequence ((D/N)X(D/N)(E/Q)Xm(D/N)Xn(Y/F) with m and n representing variable number of residues; D aspartic acid, N asparagine, E glutamic acid, Q glutamine, Y tyrosine, F phenylalanine)
Other missense mutations: segregation in family if possible + absence in 400 ethnically matched control chromosomes, if no family history absence in 400 ethnically matched control chromosomes
Linkage of haplotype for *n* ≥ 6 meioses to the FBN1 locus

*Truncating* variants were considered to be those with a *nonsense* or *frameshift* effect on the protein.

### Familial screening

If findings showed a pathogenic genetic variant, first degree relatives began a cascade screening process consisting of an exhaustive physical examination, a transthoracic echocardiography and genetic testing to screen for the same FBN-1 variant that had been found in the index case.

### Follow-up and surgery

All patients had a clinical follow-up, at intervals that depended on the clinical presentation and extent of aortic root dilation, in keeping with the recommendations of clinical guidelines. Patients with an aortic diameter larger than recommendations in guidelines (MFS diagnosis and aortic diameter > 50 mm) were referred for aortic surgery [15, 16]. Programmed procedures for TA, without aortic dissection or tear, were classified as *prophylactic surgery*. In cases of an absence of aortic regurgitation (AR), or in the presence of AR secondary to annular dilation, the options selected were *valve-sparing* aortic root replacement techniques, either the David procedure (aortic valve reimplanted in a tubular graft and reimplanted coronary ostia) or the Yacoub technique (replacement of the aortic sinuses with or without aortic annuloplasty). In other cases (an emergency situation or significant AR), the chosen procedure was concomitant valve replacement following the classic Bono-Bentall (BB) technique. In our study, the term *aortic event* included TAs with dimensions within surgical parameters and *type A aortic dissections* (TAAD). As in previous studies, because there were no patients below 18 years in our event-suffering population, a secondary analysis of the cohort was performed, excluding patients under the age of 18.

### Statistics

Qualitative variables are expressed in percentages and relationship contrasts were analysed using the chi-square (χ [2]) test or, failing that, the Fischer test. Quantitative variables are expressed as a mean ± standard deviation; their distribution was analysed with the Kolmogorov-Smirnov test and intervals were analysed using Student’s t-test for variables that followed a normal distribution, and the Mann-Whitney U for those that did not. All statistical analyses were conducted using SPSS v21 (IBM Corporation, New York, USA).

## Results

### Clinical characteristics

Of the 532 patients attended at the Hereditary Aortopathy clinic during the study period, genetic studies were requested for clinical indication and suspected MFS for a total 108 patients. Of these, FBN-1 mutations were found in 90 patients (83.33%) corresponding to 58 non-interrelated families. Table 3 contains information about the clinical characteristics of the study population, and a breakdown by age (above or below 18 years). Worthy of note are the low age of the patients studied (31.44 ± 16.92 years) and the higher proportion of male patients (55.6%); 34.4% presented MVP of any grade and 38.9% had a history of EL, which was prevalent in 61.9% of under-18 s. A high proportion of patients are receiving medical treatment with ACE inhibitors/ARBs (48.9%) or beta blockers (58.9%), with lower percentages in children, due either to intolerance or parental refusal. Of the 30% who have undergone surgical intervention, all are adults over the age of 18 who received either prophylactic surgery for TA (18.9%) or emergency surgery for TAAD (7.9%). The reason for intervention (with or without concomitant aortic repair) was MVP with severe mitral regurgitation in 3 patients (3.3%). It is noteworthy that among the prophylactic surgery group of patients, 70.6% were able to benefit from valve sparing aortic root replacement techniques (David or Yacoub). Since 2010, the year in which the Hereditary Aortopathy clinic began, prophylactic surgery has been performed on 11 patients, in 100% of cases using valve-sparing aortic root replacement techniques. Perioperative mortality among these patients was 0%. During a mean follow-up period of 31.43 ± 25.02 months, there were no deaths or emergency procedures among the patients attending consultations at our clinic.

**Table 3:** Clinical characteristics of patients with suspected MFS and FBN-1 variants

	<18 years (*n* = 21)	≥18 years (*n* = 69)	All (*n* = 90)
Age, years	9 ± 4.46	39.6 ± 11.45	31.44 ± 16.92
Sex (female)	7 (33.3%)	33 (47.8%)	40 (44.4%)
Height, cm	141.85 ± 29.95	178.6 ± 24.14	167.3 ± 30.99
Weight, kg	40.02 ± 25.01	84.92 ± 27.37	71.15 ± 33.71
FH aortic event/MFS	5 (23.8%)	20 (29%)	25 (27.8%)
Ectopia lentis	13 (61.9%)	22 (31.9%)	35 (38.9%)
Echocardiogram	21 (100%)	69 (100%)	90 (100%)
Aortic annulus, mm	18.73 ± 3.55	22.26 ± 4.56	20.94 ± 4.52
Sinuses of Valsalva, mm	30.09 ± 6.04	40.99 ± 5.93	37.51 ± 7.83
Sinotubular junction, mm	24.58 ± 5.49	34.14 ± 4.72	29.71 ± 6.97
Ascending aorta, mm	23.57 ± 6,46	34.03 ± 6,09	29.45 ± 8,1
AR (any grade)	2 (9.5%)	14 (20.3%)	16 (17.8%)
Mild AR (grade I)	2 (9.5%)	10 (14.5%)	12 (13.3%)
Moderate AR (grade II)	0 (0%)	4 (5.8%)	4 (4.4%)
Moderate to severe or severe AR (grades III-IV)	0 (0%)	0 (0%)	0 (0%)
MVP (any grade of MI)	10 (47.6%)	21 (30.4%)	31 (34.4%)
MVP – mild MI (grade I)	6 (28.6%)	16 (23.2%)	22 (24.4%)
MVP - Moderate MI (grade II)	4 (19.1%)	2 (2.9%)	6 (6.7%)
MVP - Moderate to severe MI (grade III)	0 (0%)	0 (0%)	0 (0%)
MVP – severe MI (grade IV)	0 (0%)	3 (4.3%)	3 (3.3%)
LVD (LVEF < 55%)	0 (0%)	2 (2.9%)	2 (2.2%)
Medical treatment	11 (52.4%)	58 (84.1%)	69 (76.7%)
ACE inhibitors/ARBs	2 (9.5%)	42 (60.9%)	44 (48.9%)
Beta blockers	12 (57.1%)	41 (59.4%)	53 (58.9%)
Surgical treatment	0 (0%)	27 (39.1%)	27 (30%)
Reason for surgical treatment			
TA	0 (0%)	17 (24.6%)	17 (18.9%)
TAAD	0 (0%)	7 (10.1%)	7 (7.8%)
MVP	0 (0%)	3 (4.3%)	3 (3.3%)
Type of surgical treatment			
Bono-Bentall	0 (0%)	11 (15.9%)	11 (12.2%)
Aortic valve-sparing	0 (0%)	15 (21.7%)	15 (16.7%)
Mitral valve replacement	0 (0%)	4 (5.8%)	4 (4.4%)
Modified Ghent criteria of MFS	19 (90.5%)	65 (94.2%)	84 (93.3%)
Follow-up, months	26 ± 19.52	33.41 ± 26.61	31.43 ± 25.02
Death	0 (0%)	0 (0%)	0 (0%)

*ACE inhibitors* angiotensin-converting-enzyme inhibitors, *ARBs* Angiotensin II receptor antagonists, *FH* Family history, *LVD* Left ventricle dysfunction, *LVEF* Left ventricle ejection fraction, *MI* Mitral insufficiency, *MVP* Mitral valve prolapse, *SD* Sudden death, *TA* Thoracic aorta aneurysm, *TAAD* Type A dissection

### Genetic characteristics

In the 58 families studied, 57 variants of FBN-1 were found. One patient presented with two different FBN-1 variants –c.7754 T > C and c.8176C > T-, and three a priori non-interrelated families that came from the same geographical area presented the same variant, c.6658C > T.

Of the 57 variants in the study, 25 of them (43.9%) had already been published, 23 of which had been identified as associated with MFS, while the remaining 32 (56.1%) are reported for the first time in our study (Table 4).

**Table 4:** Characteristics of FBN-1 variants (*n* = 57)

	Number (Percent)
Previously described	25 (43.9%)
Previously described and related to MFS	23 (40.4%)
De novo	11 (19.3%)
Missense	32 (56.1%)
Missense with cysteine involvement	15 (26.3%)
Nonsense	13 (22.8%)
Frameshift	8 (14.0%)
Intronic	4 (7.0%)
cbEGF-like domain	38 (66.7%)
TgfBP domain	9 (15.8%)
Pathogenic	46 (80.7%)

The majority of the genetic variants, 32, were missense (56.1%). In addition, 13 nonsense (22.8%) and 8 frameshift (14.0%) variants were found, which gave a total of 21 “*truncating*” variants. The four remaining variants affected intronic regions, which in all cases had distances lower than 3 nucleotides to the closest exon.

Of these variants, 11 were de novo (19.3%), that is to say, absence of the same variant was confirmed in both parents. In accordance with the criteria for causal FBN-1 mutations, 46 mutations (80.7%) could be considered pathogenic.

### Genotype-phenotype correlation

For 84 (93.3%) of the 90 patients with FBN-1 variants, it was possible to give a definite diagnosis of Marfan syndrome in accordance with modified Ghent criteria. The remaining 6 patients were: a 48-year-old woman with systemic characteristics and MVP but no aortopathy, whose variant (p.Met1Lle) has not been previously described and do not fulfill other criteria for causal FBN1 mutation; a 68-year-old male who only presents with TA and a variant of uncertain significance (p.Arg62His); an 8-year-old girl with EL, a systemic score < 7, a normal aortic diameter for her age and a missense variant in exon 2 of FBN-1 (p.Trp71Cys), which could therefore be classified, at least for the time being and pending possibly greater phenotypic expression during her growth, of *ectopia lentis syndrome*; a 34-year-old woman under study for coronary dissection, with borderline systemic criteria and aortic diameter, who presented a *missense* type variant in exon 44 (p.Arg1840Cys); a 8-year-old boy with EL and MVP whose variant (p.Arg2335Trp) has not been associated to aortopathy and do not fulfill other criteria for causal FBN1 mutation; and a 30-year-old male with systemic score ≥ 7 and two missense variants - one previously identified as causing MFS, p.Ile2585Thr, and the other also previously published, but with doubts to its causality, p.Arg2726Trp –, but no aortopathy, EL or proven family history.

Among patients with definite diagnosis of MFS, a direct comparison of the two types of variant most frequently found (missense vs. truncating) was performed. Patients with missense variants presented a higher prevalence of EL (56.8 vs. 8.6%, *p* < 0.001), while those who had truncating variants (nonsense or frameshift) more frequently presented with systemic criteria >7 (54.1 vs. 82.9%, *p* = 0.006) (Table 5). Of the 44 patients with missense variants, 6 had suffered an aortic event, whereas this figure was 20 of the 35 patients with truncating variants (13.6% vs. 57.1%, p < 0.001). Thus, the majority of the variants observed in the group with aortic events were *truncating* (71.4%) followed by *missense* (21.4%) and *intronic* (7.1%). On the other hand, among the population without aortic events, 67.9% had *missense* variants (Table 6). In a secondary analysis of the cohort that excluded patients below the age of 18, patients over 18 years without aortic events had a similar frequency of *truncating* or *intronic* variants (32.4%) as the patients without events of all ages (32.2%).

**Table 5:** Type of FBN-1 variant and phenotype in patients with diagnosis of MFS

	Missense (*n* = 44)	Truncating (*n* = 35)	p
Mitral valve prolapse *n* (%)	16 (36.4%)	9 (25.7%)	0.312
Ectopia lentis, *n* (%)	25 (56.8%)	3 (8.6%)	<0.001
Systemic ≥7, *n* (%)	23 (54.1%)	29 (82.9%)	0.006
Family history, *n* (%)	15 (34.1%)	8 (22.9%)	0.275
Aortic event, *n* (%)	6 (13.6%)	20 (57.1%)	<0.001
TA, *n* (%)	5 (11.4%)	13 (37.1%)	0.007
TAAD, *n* (%)	1 (2.3%)	7 (20.0%)	0.009

*MVP* Mitral valve prolapse, *SD* Sudden death, *TA* Thoracic aorta aneurysm, *TAAD* Type A dissection

**Table 6:** Characteristics of patients with MFS and aortic events compared to patients without aortic events

	TA in surgical range (*n* = 20)		Aortic dissection (*n* = 8)		Total cohort with event (*n* = 28)		Patients without aortic events (*n* = 56)	Patients over 18 without aortic events (*n* = 37)
		(Median age at event (range))		(Median age at event (range))		(Median age at event (range))		
Male, *n* (%)	11 (55%)	(39 (20–55))	5 (62.5%)	(43 (23–50))	16 (57.1%)	(39.5 (20–55))	32 (57.1%)	19 (51.4%)
Patients with missense mutation, *n* (%)	5 (25%)	(40 (38–43))	1 (12.5%)	(48 (48–48))	6 (21.4%)	(40.5 (38–48))	38 (67.9%)	25 (67.5%)
Patients with truncating mutation, *n* (%)	13 (65%)	(36 (20–55))	7 (87.5%)	(43 (23–50))	20 (71.4%)	(39.5 (20–55))	15 (26.8%)	9 (24.3%)
Patients with intronic mutation, *n* (%)	2 (10%)	(33.5 (24–43))	–	–	2 (7.1%)	(33.5 (24–43))	3 (5.4%)	3 (8.1%)

Events appeared at slightly earlier ages in patients with *truncating* variants than in those with *missense*, although there were no statistically significant differences (41.33 ± 3.77 vs. 37.5 ± 9.62 years, *p* = 0.162). The median age at time of the aortic event was 40.5, 39.5 and 33.5 years for patients with *missense, truncating* or *intronic* variants respectively. The analysis by age for each strata showed 5 patients with aortic events (3 male and 2 female) before the age of 30, of whom 4 (80%) had *truncating* and 1 (20%) *intronic* variants. Before the age of 40, 13 patients (8 male, 5 female) had presented an aortic event, in 2 cases with a *missense* type variant (15.4%), 10 with a *truncating* variant (76.9%) and 1 with an intronic variant (7.7%). The 2 patients with an aortic event before the age of 40 and a variant that was neither *truncating* nor *intronic* were: 1) A male, who underwent surgery at age 38 for TA, with systemic criteria ≥7 and variant p.Gly343Arg in the TGFBP domain of FBN-1, described in previous studies but catalogued by them as “of uncertain significance” [23], who also has a 41 year old brother with TA (aortic diameter of 51 mm) and identification of the same variant; and 2) a female, also operated at 38 years for TA, with systemic criteria and EL, with variant p.Cys1097Tyr in the cb-EGF-like domain of exon 26 of FBN-1 (Additional file 1: Table S1).

In the same way, variant type and the age of event were also compared for patients with prophylactic aortic surgery vs. dissection (Table 6). Patients with dissection (*n* = 8) less frequently, but not significantly, had *missense* type variants in comparison with patients undergoing prophylactic aortic surgery (*n* = 20), (12.5 vs. 25%; *p* = 0.466). Among the patients with truncating mutation and aortic event, the median age for TAAD was 43 years (*n* = 7), and for TA was 36 years (*n* = 13).

## Discussion

To our knowledge, this study represents the largest series of patients with MFS and mutations in FBN-1 in the Mediterranean population to date. On the other hand, it contributes valuable knowledge about the phenotypic expression of new, not previously described variants.

In this study, we describe the clinical characteristics of 90 patients with FBN-1 variants and suspected MFS. As in classic studies of MFS, the clinical heterogeneity of our patients is worthy of note: we found cases ranging from mild forms with aortic diameters that were normal or only slightly larger than normal, to forms with a higher phenotypical expression with or without aortic events [24, 25].

In our study, 38.9% had a history of EL and 34.4% of MVP, frequencies that are slightly lower than those published in other large series of MFS patients [26, 27].

In our sample, 30% underwent surgical intervention, either prophylactic for TA (18.9%) or emergency for TAAD (7.9%). Recently, in their prospective study that included 146 MFS patients, David et al. showed that valve-sparing aortic root replacement techniques in Marfan syndrome patients are associated with low rates of valve-related complications in long-term follow-up [28]. In our experience, over half of all of the operated patients benefitted from these techniques; this figure rises to 100% when considering only the patients attended since the Hereditary Aortopathy clinic was opened in 2010. In our opinion, close collaboration with a Cardiac Surgery service that has special training in these techniques may be a key factor to achieving these figures.

The FBN-1 gene (>200 kb), composed of 65 exons, is found on the long arm of chromosome 15 (15q15-q21.1) and codifies 2871 amino acids. It has a modular structure that includes 47 six-cysteine EGF-like domains, 7 eight-cysteine TGF-binding proteins, and a proline-rich region [29]. Almost 3000 variants related with MFS have been described, according to the most extensive FBN-1 mutation database [30], although it is likely that this number is currently higher as the database has not recorded many of the variants identified worldwide [12]. Our study provides clinical information about patients with 25 of these previously published variants, and adds another 32 to scientific knowledge, with a detailed phenotype description and, in many cases, more than one individual affected by the variant.

Most of the variants found in our study were *missense* type (56.1%), followed by *truncating* (36.8%) and *intronic* (7.1%). Similar percentages have been found in previously published large series such as those of *Faivre* et al. (56%, 33% y 11%, respectively), and of *Baudhuin* et al. (53%, 33% y 13%, respectively) [13].

Although there are numerous previously published studies on FBN-1 genotyping, establishing broad, solid conclusions about the phenotype-genotype connection has classically proved difficult. Among the few reproducible phenotypic findings dependent on genotype is EL, which has been associated with *missense* type mutations (particularly those that affect cysteine residues), and the feared “neonatal” MFS, related with mutations in the central region of the gene (exons 24–32) [31, 32]. On the other hand, it was previously suspected that patients with truncating mutations could have a slower or milder progression of the disorder [33], and *Baudhuin* et al. were the first to link this type of variant with a higher percentage of aortic events and a lower presentation age [13]. Our data supports this observation, with 71.4% of aortic events occurring in patients with *truncating* variants and a median age for TAAD of 43 years (versus 21.4% of aortic events among patients with *missense* variants and a median age of 48 years for TAAD). *Franken* et al. recently confirmed this worse prognosis in a prospective study of 570 MFS patients in which those with *haploinsufficiency* of FBN-1 presented a 2.4 times higher risk for the combined endpoint of cardiovascular death and dissection [14]. Furthermore, it has even been suggested that the patients with this genetic defect in FBN-1 seem to respond better to treatment with Losartan, with lower aortic root growth rates [34].

Therefore, the finding of one or other type of variant has prognostic implications and should guide clinical management. Some individuals could also benefit from closer follow-up, recommendations for an appropriate lifestyle, and a more vigorous medical treatment that helps reduce the progression of this disorder towards potentially fatal aortic events. Genetic testing was also of great help in identification of under-18 s with systemic findings suggestive of MFS (particularly those who do not fully meet the criteria) who did not have vascular findings at the time of the initial screening and could therefore benefit from an annual reassessment based on an echocardiogram to detect a potentially swift onset of aortic complications.

In our opinion, the existence of hereditary aortopathy units is of the utmost importance. Such units consist of cardiologists, cardiac surgeons, ophthalmologists, orthopaedic specialists and geneticists who facilitate the correct management of patients who suffer the complex pathology of a disorder such as MFS and other patients affected by fibrillin-related disorders who do not strictly meet MFS criteria. Such teams can adapt to the specific clinical and genetic characteristics of each patient, and can also make sense of the enormous amount of information obtained from genetic studies [35].

## Conclusions

Our Hereditary Aortopathy clinic was able to identify 90 patients with suspected MFS and FBN-1 variants, more than half of which had not previously been described. Although phenotype varied widely, patients with confirmed MFS and *truncating* variants presented a higher proportion of aortic events. Genetic findings could, therefore, have importance in risk stratification and in the clinical management of suspected MFS patients.

## Additional file

Additional file 1: Table S1. Variants in FBN-1 and phenotypic characteristics of the carrier patients. (DOCX 79 kb)

## Abbreviations

AR: Aortic regurgitation
BB: Bono-Bental
EL: Ectopia lentis
MAD: Mild aortic dilatation
MFS: Marfan syndrome
MVP: Mitral valve prolapse
SD: Sudden death
TA: Thoracic aorta aneurysm
TAAD: Type A aortic dissection

## References

[1] Giudicessi JR, Iftikhar JK, Ackerman M (2017). Precision cardiovascular medicine: state of genetic testing. Mayo Clin Proc.

[2] Proost D, Vandeweyer G, Meester JA, Salemink S, Kempers M, Ingram C (2015). Performant mutation identification using targeted next-generation sequencing of 14 thoracic aortic aneurysm genes. Hum Mutat.

[3] Sinha KP, Goldberg H (1958). Marfan's syndrome: a case with complete dissection of the aorta. Am Heart J.

[4] Groth KA, Hove H, Kyhl K, Folkestad L, Gaustadnes M, Vejlstrup N (2015). Prevalence, incidence, and age at diagnosis in Marfan syndrome. Orphanet J Rare Dis.

[5] Judge DP, Dietz HC. Marfan’s syndrome. Lancet. 2005;366:1965–76. 10.1016/S0140-6736(05)67789-6 PMC1513064 16325700

[6] De Paepe A, Devereux RB, Dietz HC, Hennekam RC, Pyeritz RE (1996). Revised diagnostic criteria for the Marfan syndrome. Am J Med Genet.

[7] Lipscomb KJ, Clayton-Smith J, Harris R (1997). Evolving phenotype of Marfan's syndrome. Arch Dis Child.

[8] Lee B, Godfrey M, Vitale E, Hori H, Mattei MG, Sarfarazi M (1991). Linkage of Marfan syndrome and a phenotypically related disorder to two different fibrillin genes. Nature.

[9] Dietz HC, Cutting GR, Pyeritz RE, Maslen CL, Sakai LY, Corson GM (1991). Marfan syndrome caused by a recurrent de novo missense mutation in the fibrillin gene. Nature.

[10] Sakai LY, Keene DR, Renard M, De Backer J (2016). FBN1: The disease-causing gene for Marfan syndrome and other genetic disorders. Gene.

[11] Loeys B, De Backer J, Van Acker P, Wettinck K, Pals G, Nuytinck L (2004). Comprehensive molecular screening of the FBN1 gene favors locus homogeneity of classical Marfan syndrome. Hum Mutat.

[12] Loeys BL, Dietz HC, Braverman AC, Callewaert BL, De Backer J, Devereux RB (2010). The revised Ghent nosology for the Marfan syndrome. J Med Genet.

[13] Baudhuin LM, Kotzer KE, Lagerstedt SA (2015). Increased frequency of FBN1 truncating and splicing variants in Marfan syndrome patients with aortic events. Genet Med.

[14] Franken R, Groenink M, de Waard V, Feenstra HM, Scholte AJ, van den Berg MP (2016). Genotype impacts survival in Marfan syndrome. Eur Heart J.

[15] Stheneur C, Tubach F, Jouneaux M, Roy C, Benoist G, Chevallier B, Boileau C, Jondeau G (2014). Study of phenotype evolution during childhood in Marfan syndrome to improve clinical recognition. Genet Med.

[16] Hiratzka LF, Bakris GL, Beckman JA, Bersin RM, Carr VF, Casey DE (2010). 2010 ACCF/AHA/AATS/ACR/ASA/SCA/SCAI/SIR/STS/SVM guidelines for the diagnosis and management of patients with thoracic aortic disease. Circulation.

[17] Erbel R, Aboyans V, Boileau C, Bossone E, Bartolomeo RD, Eggebrecht H (2014). 2014 ESC guidelines on the diagnosis and treatment of aortic diseases: document covering acute and chronic aortic diseases of the thoracic and abdominal aorta of the adult. The task force for the diagnosis and treatment of aortic diseases of the European Society of Cardiology (ESC). Eur Heart J.

[18] Devereux RB, de Simone G, Arnett DK, Best LG, Boerwinkle E, Howard BV (2012). Normal limits in relation to age, body size and gender of two-dimensional echocardiographic aortic root dimensions in persons ≥15 years of age. Am J Cardiol.

[19] Gautier M, Detaint D, Fermanian C, Aegerter P, Delorme G, Arnoult F (2010). Nomograms for aortic root diameters in children using two-dimensional echocardiography. Am J Cardiol.

[20] Quiñones MA, Otto CM, Stoddard M, Waggoner A, Zoghbi WA (2002). Doppler quantification task force of the nomenclature and standards Committee of the American Society of echocardiography. Recommendations for quantification of Doppler echocardiography: a report from the Doppler quantification TaskForce of the nomenclature and standards committee of the American Societyof echocardiography. J Am Soc Echocardiogr.

[21] Richards S, Aziz N, Bale S, Bick D, Das S, Gastier-Foster J (2015). ACMG laboratory quality assurance committee. Standards and guidelines for the interpretation of sequence variants: a joint consensus recommendation of the American College of Medical Genetics and Genomics and the Association for Molecular Pathology. Genet Med.

[22] Den Dunnen JT, Dalgleish R, Maglott DR, Hart RK, Greenblatt MS, McGowan-Jordan J, et al. Hum Mutat. 2016;37:564-569. Available in: http://varnomen.hgvs.org. Accessed 1 May 2017. 10.1002/humu.22981 26931183

[23] Tjeldhorn L, Rand-Hendriksen S, Gervin K, Brandal K, Inderhaug E, Geiran O (2006). Rapid and efficient FBN1 mutation detection using automated sample preparation and direct sequencing as the primary strategy. Genet Test.

[24] Bruno L, Tredici S, Mangiavacchi M, Colombo V, Mazzotta GF, Sirtori CR (1984). Cardiac, skeletal, and ocular abnormalities in patients with Marfan's syndrome and in their relatives. Comparison with the cardiac abnormalities in patients with kyphoscoliosis. Br Heart J.

[25] Keane MG, Pyeritz RE (2008). Medical management of Marfan syndrome. Circulation.

[26] Tsipouras P, Del Mastro R, Sarfarazi M, Lee B, Vitale E, Child AH (1992). Genetic linkage of the Marfan syndrome, ectopia lentis, and congenital contractural arachnodactyly to the fibrillin genes on chromosomes 15 and 5. The international Marfan syndrome collaborative study. N Engl J Med.

[27] Rybczynski M, Mir TS, Sheikhzadeh S, Bernhardt AM, Schad C, Treede H (2010). Frequency and age-related course of mitral valve dysfunction in the Marfan syndrome. Am J Cardiol.

[28] David TE, David CM, Manlhiot C, Colman J, Crean AM, Bradley T (2015). Outcomes of aortic valve-sparing operations in Marfan syndrome. J Am Coll Cardiol.

[29] Ramachandra CJ, Mehta A, Guo KW, Wong P, Tan JL, Shim W (2015). Molecular pathogenesis of Marfan syndrome. Int J Cardiol.

[30] Collod-Beroud G, Le Bourdelles S, Ades L, Ala-Kokko L, Booms P, Booms P, et al. Update of the UMD-FBN1 mutation database and creation of an FBN1 polymorphism database. Hum Mutat. 2003;22:199–208. Available in: http://www.umd.be/FBN1/. Last update 28 August 2014. Accessed 1 May 2017. 10.1002/humu.10249 12938084

[31] Faivre L, Collod-Beroud G, Loeys BL, Child A, Binquet C, Gautier E (2007). Effect of mutation type and location on clinical outcome in 1,013 probands with Marfan syndrome or related phenotypes and FBN1 mutations: an international study. Am J Hum Genet.

[32] Schrijver I, Liu W, Brenn T, Furthmayr H, Francke U (1999). Cysteine substitutions in epidermal growth factor-like domains of fibrillin-1: distinct effects on biochemical and clinical phenotypes. Am J Hum Genet.

[33] Rand-Hendriksen S, Tjeldhorn L, Lundby R, Semb SO, Offstad J, Andersen K (2007). Search for correlations between FBN1 genotype and complete Ghent phenotype in 44 unrelated Norwegian patients with Marfan syndrome. Am J Med Genet A.

[34] Franken R, den Hartog AW, Radonic T, Micha D, Maugeri A, van Dijk FS (2015). Beneficial outcome of losartan therapy depends on type of FBN1 mutation in Marfan syndrome. Circ Cardiovasc Genet.

[35] Barriales-Villa R, Gimeno-Blanes JR, Zorio-Grima E, Ripoll-Vera T, Evangelista-Masip A, Moya-Mitjans A (2016). Plan of action for inherited cardiovascular diseases: synthesis of recommendations and action algorithms. Rev Esp Cardiol.

