# Supplementary material for: The importance of genotype-phenotype correlation in the clinical management of Marfan syndrome
Source: Orphanet J Rare Dis. 2018 Jan 22;13:16. doi: 10.1186/s13023-017-0754-6 (PMC5778633; doi:10.1186/s13023-017-0754-6)
Supplement: Additional file 1: Table S1. — Variants in FBN-1 and phenotypic characteristics of the carrier patients. (DOCX 79 kb) [file 13023_2017_754_MOESM1_ESM.docx]

**Addtional file 1 Table S1. –** Variants in FBN-1 and phenotypic characteristics of the carrier patients.

| **Genetic variant** | | | | | | | | **Phenotypic characteristics** | | | | | | | **MFS** |
| --- | --- | --- | --- | --- | --- | --- | --- | --- | --- | --- | --- | --- | --- | --- | --- |
| **Meaning** | **Type** | **Exon /**  **Intron** | **Nucleotide change** | **Protein change** | **Domain** | **Inherit-ance** | **Described** | **Nº family members** | **Sex** | **Age** | **Vascular** | **Systemic ≥7** | **Ocular** | **Family history** |  |
| Missense | Sub | Exon 1 | c.3G>A | p.Met1LIe | Signal peptide | Mother | No | 1 | F | 48 | MVP | Yes | EL, SM |  | No |
| Missense | Sub | Exon 2 | c.175T>C | p.Cys59Arg | 4-cys motif LTBP-like | Father | Yes^6^★ | 1 | M | 39 | MAD, MVP | Yes | EL | SD (M 40y) | Yes |
| Missense | Sub | Exon 2 | c.185G>A | p.Arg62His | 4-cys motif LTBP-like | ND | Yes^14^ ★ | 1 | M | 68 | TA | No | No |  | No |
| Missense | Sub | Exon 2 | c.213G>C | p.Trp71Cys | 4-cys motif LTBP-like | De novo | No | 1 | F | 8 | No | No | EL |  | No |
| Nonsense | Sub | Exon 4 | c.349C>T | p.Gln117* | EGF-like #02 | ND | No | 2 | M | 31 | TA (BB 20y) | Yes | No |  | Yes |
|  |  |  |  |  |  |  |  |  | M | 30 | TA (Yacoub 30y) | Yes | No |  | Yes |
| Nonsense | Sub | Exon 5 | c.493C>T | p. Arg165* | EGF-like#03 | ND | Yes ^12^ | 1 | F | 33 | MAD, MVP | No | No | TAAD (F 42y) | Yes |
| Nonsense | Sub | Exon 6 | c.643C>T | p.Arg215* | Hybrid module #01 | ND | Yes^1^ | 2 | F | 42 | TA (David 40 y) | Yes | EL, SM |  | Yes |
|  |  |  |  |  |  |  |  |  | M | 15 | MAD, MVP | Yes | No |  | Yes |
| Frameshift | Ins | Exon 8 | c.950insC | p.Pro317ProfsX30 | cb EGF-like #02 | Father | No | 1 | M | 45 | TA (David 39 y) | No | SM | TAAD (M 50y) | Yes |
| Missense | Sub | Exon 9 | c.1027G>A | p.Gly343Arg | TGFBP#01 | Mother | Yes^2^ | 2 | M | 45 | TA (BB 38y), MVP | Yes | No |  | Yes |
|  |  |  |  |  |  |  |  |  | M | 41 | TA, MVP | Yes | EL |  | Yes |
| Missense | Sub | Exon 9 | c.1097G>C | p.Trp366Ser | TGFBP#01 | Father | No | 2 | M | 17 | MAD | Yes | No | SD (M 46y) | Yes |
|  |  |  |  |  |  |  |  |  | F | 11 | MVP | No | No | SD (M 46y) | Yes |
| Intronic | Sub | Exon  9-10 | c.1148-1G>A |  | TGFBP#01 | ND | No | 2 | F | 45 | MAD | Yes | EL | SD (M 42y) | Yes |
|  |  |  |  |  |  |  |  |  | F | 22 | MAD, MVP | Yes | No | SD (M 42y) | Yes |
| Nonsense | Sub | Exon 13 | c.1533C>G | p.Tyr511* | cb EGF-like #03 | ND | No | 1 | F | 43 | TA (Yacoub 40y) | Yes | No |  | Yes |
| Missense | Sub | Exon 14 | c.1792T>C | p.Cys598Arg | cb EGF-like #05 | Mother | No | 1 | M | 15 | No | No | EL | TA (F 40y) | Yes |
| Missense | Sub | Exon 16 | c.1849T>C | p.Cys617Arg | cb EGF-like #06 | ND | No | 1 | F | 19 | MAD, MVP, osAID | Yes | EL |  | Yes |
| Missense | Sub | Exon 18 | c.2177A>C | p.Glu726Ala | cb EGF-like #07 | ND | No | 1 | M | 51 | MAD | Yes | EL |  | Yes |
| Missense | Sub | Exon 18 | c.2223A>G | p.Asn741Ser | cb EGF-like #07 | ND | No | 2 | M | 43 | MAD | No | EL |  | Yes |
|  |  |  |  |  |  |  |  |  | M | 18 | MAD, BAV, MVP | No | EL |  | Yes |
| Missense | Sub | Exon 18 | c.2242T>C | p.Cys748Arg | cb EGF-like #07 | Mother | No | 1 | F | 45 | TA (Yacoub 43y), MVP | Yes | EL | TAAD (F 21y, M 24y), SD (M 50y) | Yes |
| Missense | Sub | Exon 18 | c.2258G>T | p.Gly753Val | cb EGF-like #07 | De novo | No | 1 | M | 14 | MAD | Yes | EL |  | Yes |
| Missense | Sub | Exon 19 | c.2375G>A | p.Cys792Tyr | cb EGF-like #08 | Father | Yes^13^ | 1 | F | 41 | MAD, MVP | Yes | EL | TAAD (M 52y), SD (M 7y) | Yes |
| Nonsense | Sub | Exon 20 | c.2438C>G | p.Ser813* | cb EGF-like #09 | De novo | Yes^1^ | 1 | F | 9 | MAD | Yes | No |  | Yes |
| Missense | Sub | Exon 20 | c.2495G>A | p.Cys832Tyr | cb EGF-like #09 | ND | Yes^4^ | 2 | M | 41 | MAD | Yes | EL |  | Yes |
|  |  |  |  |  |  |  |  |  | F | 1 | MAD | No | EL |  | Yes |
| Nonsense | Sub | Exon 21 | c.2581C>T | p.Arg861* | Hybrid motif #02 |  | Yes^4^ | 1 | F | 50 | TAAD (David 48 y) | Yes | No |  | Yes |
| Missense | Sub | Exon 23 | c.2740 T>A | p.Cys914Ser | cb EGF-like #10 | ND | Yes^7^ | 9 | F | 65 | MAD | No | SM |  | Yes |
|  |  |  |  |  |  |  |  |  | M | 63 | MAD | No | No |  | Yes |
|  |  |  |  |  |  |  |  |  | F | 57 | No | No | SM |  | Yes |
|  |  |  |  |  |  |  |  |  | F | 38 | MAD | No | EL |  | Yes |
|  |  |  |  |  |  |  |  |  | M | 31 | MAD | No | No |  | Yes |
|  |  |  |  |  |  |  |  |  | M | 27 | MAD | No | EL |  | Yes |
|  |  |  |  |  |  |  |  |  | M | 10 | MAD | No | EL |  | Yes |
|  |  |  |  |  |  |  |  |  | M | 8 | MAD | No | EL |  | Yes |
|  |  |  |  |  |  |  |  |  | F | 7 | MAD | No | EL |  | Yes |
| Missense | Sub | Exon 26 | c.3290G>A | p.Cys1097Tyr | cb EGF-like #12 | ND | No | 1 | F | 46 | TA (BB 38y) | Yes | EL |  | Yes |
| Nonsense | Sub | Exon 27 | c.3373 C>T | p.Arg1125* | cb EGF-like #13 | ND | Yes^3^ | 3 | F | 48 | TA (BB 25y) | Yes | SM | SD (M 33y) | Yes |
|  |  |  |  |  |  |  |  |  | M | 21 | MAD | Yes | EL | SD (M 33y) | Yes |
|  |  |  |  |  |  |  |  |  | F | 17 | MAD | Yes | EL, SM | SD (M 33y) | Yes |
| Nonsense | Sub | Exon 27 | c.3397G>T | p.Glu1133* | cb EGF-like #13 | ND | No | 1 | F | 43 | TAAD (BB 43 y) | Yes | SM |  | Yes |
| Frameshift | Del | Exon 27 | c.3439delT | p.Ser1147fs*1161 | cb EGF-like #13 | Father | No | 1 | F | 42 | TA (David 36 y), MVP | Yes | No | SD (M 24y), SD (F 45y) | Yes |
| Frameshift | Del | Exon 31 | c.3848_3849delAG | Glu1283fs*1284 | cb EGF-like #17 | ND | No | 2 | F | 46 | MAD, MVP (PMVR 33 y) | Yes |  |  | Yes |
|  |  |  |  |  |  |  |  |  | M | 25 | MAD | Yes | SM |  | Yes |
| Missense | Sub | Exon 32 | c.3977G>C | p.Cys1326Ser | cb EGF-like #18 | De novo | No | 1 | M | 3 | MAD, MVP | Yes | EL |  | Yes |
| Intronic | Sub | Exon 33-34 | c.4210+1 G>A |  | cb EGF-like #20 | ND | No | 1 | M | 45 | TA (Yacoub 43y) | Yes | EL |  | Yes |
| Nonsense | Sub | Exon 34 | c.4255C>T | p.Gln1419* | cb EGF-like #20 | Mother | No | 2 | M | 57 | TA (BB 50y) | Yes | No |  | Yes |
|  |  |  |  |  |  |  |  |  | F | 31 | MAD | Yes | No |  | Yes |
| Missense | Sub | Exon 34 | c.4283G>C | p.Arg1428Pro | cb EGF-like #20 | De novo | No | 1 | M | 7 | MAD | No | EL |  | Yes |
| Missense | Sub | Exon 37 | c.4588C>T | p.Arg1530Cys | TGFBP#04 | ND | Yes^8^ | 2 | F | 61 | MAD MVP (MVrep + Yacoub 55y) | No | No |  | Yes |
|  |  |  |  |  |  |  |  |  | F | 27 | MAD, MVP | Yes | EL |  | Yes |
| Frameshift | InsDel | Exon 37 | c.4595_6delGAinsT | p.Gly1532Valfs*49 | TGFBP#04 | ND | No | 3 | M | 32 | TA (David 27 y) | Yes | No |  | Yes |
|  |  |  |  |  |  |  |  |  | M | 7 | MAD | Yes | No |  | Yes |
|  |  |  |  |  |  |  |  |  | M | 4 | MAD | Yes | No |  | Yes |
| Frameshift | Del | Exon 42 | c.5266_5278del | p.Val1756*fs169 | TGFBP#05 | ND | No | 2 | M | 53 | TAAD (BB 31 y) | No | No |  | Yes |
|  |  |  |  |  |  |  |  |  | M | 25 | MAD, MVP | Yes | No |  | Yes |
| Missense | Sub | Exon 43 | c.5345G>T | p.Cys1782Phe | cb EGF-like #25 | De novo | No | 1 | M | 8 | MAD | Yes | EL |  | Yes |
| Missense | Sub | Exon 44 | c.5518C>T | p.Arg1840Cys | cb EGF-like #26 | Father | No | 1 | F | 34 | SCAD | No | No |  | No |
| Frameshift | Ins | Exon 45 | c.5581insA | p.Ser1861fs*1866 | cb EGF-like #26 | ND | No | 1 | M | 39 | TA (David 33 y) | Yes | No |  | Yes |
| Missense | Sub | Exon 46 | c.5683T>C | p.Cys1895Arg | cb EGF-like #28 | ND | Yes ^5^ | 1 | M | 23 | MAD | Yes | EL |  | Yes |
| Intronic | Sub | Exon 46-47 | c.5788+1G>T |  | cb EGF-like #29 | ND | Yes^17^ | 1 | F | 32 | TA, MVP (Yacoub + MVrep 24 y) | Yes | EL |  | Yes |
| Missense | Sub | Exon 47 | c.5788G>A | p.Asp1930Asn | cb EGF-like #29 | ND | Yes^4^ | 1 | F | 14 | MAD, MVP | Yes | EL |  | Yes |
| Missense | Sub | Exon 47 | c.5834G>A | p.Gly1945Asp | cb EGF-like #29 | Mother | No | 2 | F | 34 | MAD | No | No | SD (M 50y) | Yes |
|  |  |  |  |  |  |  |  |  | M | 27 | MAD, MVP | Yes | SM | SD (M 50y) | Yes |
| Missense | Sub | Exon 52 | c.6392G>A | p.Cys2131Tyr | cb EGF-like #32 | De novo | No | 1 | M | 10 | MAD, MVP | Yes | No |  | Yes |
| Nonsense | Sub | Exon 54 | c.6658C>T | p.Arg2220* | cb EGF-like #34 | Mother | Yes^1^ | 1 | F | 51 | TA (David 43 y) BTD (45y) | No | No | SD (F 40y) | Yes |
| Nonsense | Sub | Exon 54 | c.6658C>T | p.Arg2220* | cb EGF-like #34 | ND | Yes^1^ | 1 | F | 37 | BTD (31 y) TA (David 34y) | Yes | No |  | Yes |
| Nonsense | Sub | Exon 54 | c.6658C>T | p.Arg2220* | cb EGF-like #34 | De novo | Yes^1^ | 1 | M | 9 | MAD | Yes | SM |  | Yes |
| Missense | Sub | Exon 54 | c.6661T>C | p.Cys2221Arg | cb EGF-like #34 | ND | Yes^11^ | 2🟅 | M | 40 | TA | Yes | No | SD (M 32y) | Yes |
|  |  |  |  |  |  |  |  |  | F | 37 | MAD, MVP | Yes | No | SD (M 32y) | Yes |
| Missense | Sub | Exon 55 | c.6797C>G | p.Cys2265Trp | cb EGF-like #35 | ND | No | 1 | M | 43 | MAD | Yes | No |  | Yes |
| Missense | Sub | Exon 57 | c.7003C>T | p.Arg2335Trp  (homocigosis) | TGFBP #07 | Father & Mother | Yes^9^ | 1 | M | 8 | MVP | No | EL |  | No |
| Frameshift | Del | Exon 57 | c.7039_7040delAT | p.Met2347Valfs*19 | TGFBP #07 | Father | Yes^16^ | 1 | M | 50 | TAAD (Yacoub 50y) | Yes | No | TAAD (M 64y) | Yes |
| Intronic | Sub | Exon 57-58 | c.7204+01 G>A |  | TGFBP #07 | Father | No | 1 | M | 36 | MAD, MVP | Yes | EL, SM |  | Yes |
| Frameshift | Del | Exon 58 | c.7276_7278delCAT | p.His2426del | cb EGF-like #37 | ND | No | 3 | M | 58 | TA (Yacoub 55y) | No | No |  | Yes |
|  |  |  |  |  |  |  |  |  | M | 58 | MAD, DCM | Yes | No |  | Yes |
|  |  |  |  |  |  |  |  |  | M | 24 | MAD, MVP | No | No |  | Yes |
| Missense | Sub | Exon 62 | c.7754T>C | p.Ile2585Thr | cb EGF-like #41 | De novo | Yes^4^ | 1 | M | 30 | MVP | Yes | No |  | No |
| Missense | Sub | Exon 65 | c.8176C>T | p.Arg2726Trp | FibuCTDIII-like motif | De novo | Yes ^15^ |  |  |  |  |  |  |  |  |
| Missense | Sub | Exon 63 | c.7828G>A | p.Glu2610Lys | cb EGF-like #42 | De novo | Yes^4^ | 1 | M | 23 | MAD | Yes | EL |  | Yes |
| Nonsense | Sub | Exon 63 | c.7977C>A | p.Cys2659* | cb EGF-like #43 | ND | Yes^10^ | 4 | M | 55 | TAAD (BB 40 y), MVP | Yes | No |  | Yes |
|  |  |  |  |  |  |  |  |  | M | 53 | TAAD (BB 43 y), BTD (48y) | Yes | No |  | Yes |
|  |  |  |  |  |  |  |  |  | M | 48 | TAAD, (BB 23y), BTD (31y), MVP (PMVR 40 y) | Yes | No |  | Yes |
|  |  |  |  |  |  |  |  |  | F | 44 | MAD, MVP | Yes | No |  | Yes |
| Missense | Sub | Exon 65 | c.8609T>G | p.Leu2870Arg | FibuCTDIII-like motif | Mother | No | 5 | F | 59 | MAD | No | No | TAAD (F 72y) | Yes |
|  |  |  |  |  |  |  |  |  | F | 55 | TAAD (BB 48 y), MVP | Yes | No | TAAD (F 72y) | Yes |
|  |  |  |  |  |  |  |  |  | F | 52 | MAD | Yes | SM | TAAD (F 72y) | Yes |
|  |  |  |  |  |  |  |  |  | F | 28 | No | No | SM | TAAD (F 72y) | Yes |
|  |  |  |  |  |  |  |  |  | M | 19 | MAD | No | No | TAAD (F 72y) | Yes |

**BAV=** Bicuspid aortic valve**; BB =** Bono-Bentall; **DCM =** Dilated cardiomyopathy; **Del =** Deletion; **EL =** Ectopia lentis; **F =** Female; **Ins =** Insertion; **InsDel =** Insertion + deletion; **M =** Male**;**  **MAD =** Mild aortic dilatation; **MFS** = Diagnosis of Marfan Syndrome according to the Ghent Criteria of 2010; **MVP =** Mitral valve prolapse; **MVrep =** Mitral valve repair; **osASD =** Ostium secundum atrial septal defect; **PMVR =** Prosthetic mitral valve replacement; **SCAD =** Spontaneous coronary artery dissection; **SM =** Severe myopia; **Sub =** Substitution; **TA =** Thoracic aorta aneurysm; **TAAD** = Type A dissection; **TBAD =** Type B dissection; **y** = years.

★ Previously described variants, but not proven to cause MFS.

🟅 Both patients, siblings with a common family history of early SD (father at 32 years), present a mutation in COL5A1, p.Gln126His, of unknown pathogenicity.

1. Matsukawa R, Iida K, Nakayama M, Mukai T, Okita Y, Ando M, Takamoto S, Nakajima N, Morisaki H, Morisaki T. Eight novel mutations of the FBN1 gene found in Japanese patients with Marfan syndrome. Hum Mutat. 2001;17:71-2.

2. Tjeldhorn L, Rand-Hendriksen S, Gervin K, Brandal K, Inderhaug E, Geiran O, Paus B. Rapid and efficient FBN1 mutation detection using automated sample preparation and direct sequencing as the primary strategy. Genet Test. 2006;10:258-64.

3. Rommel K, Karck M, Haverich A, von Kodolitsch Y, Rybczynski M, Müller G, Singh KK, Schmidtke J, Arslan-Kirchner M. Identification of 29 novel and nine recurrent fibrillin-1 (FBN1) mutations and genotype-phenotype correlations in 76 patients with Marfan syndrome. Hum Mutat. 2005;26:529-39.

4. Liu WO, Oefner PJ, Qian C, Odom RS, Francke U. Denaturing HPLC-identified novel FBN1 mutations, polymorphisms, and sequence variants in Marfan syndrome and related connective tissue disorders. Genet Test. 1998;1:237-42.

5. Oh MR, Kim JS, Beck NS, Yoo HW, Lee HJ, Kohsaka T, Jin DK. Six novel mutations of the fibrillin-1 gene in Korean patients with Marfan syndrome. Pediatr Int. 2000;42:488-91.

6. Wei X, Ju X, Yi X, Zhu Q, Qu N, Liu T, Chen Y, Jiang H, Yang G, Zhen R, Lan Z, Qi M, Wang J, Yang Y, Chu Y, Li X, Guang Y, Huang J. Identification of sequence variants in genetic disease-causing genes using targeted next-generation sequencing. PLoS One. 2011;6:e29500.

7. Stheneur C, Collod-Béroud G, Faivre L, Buyck JF, Gouya L, Le Parc JM, Moura B, Muti C, Grandchamp B, Sultan G, Claustres M, Aegerter P, Chevallier B, Jondeau G, Boileau C. Identification of the minimal combination of clinical features in probands for efficient mutation detection in the FBN1 gene. Eur J Hum Genet. 2009;17:1121-8.

8. Shentu XC, Tang XJ, Ye PP, Jin CF, Wang W, Yao K. Computer construction and analysis of protein models of mutant fibrillin-1 gene in Marfan's síndrome. Zhonghua Yan Ke Za Zhi. 2009;45:699-702.

9. Robinson PN, Booms P, Katzke S, Ladewig M, Neumann L, Palz M, Pregla R, Tiecke F, Rosenberg T. Mutations of FBN1 and genotype-phenotype correlations in Marfan syndrome and related fibrillinopathies. Hum Mutat. 2002;20:153-61.

10. Magyar I, Colman D, Arnold E, Baumgartner D, Bottani A, Fokstuen S, Addor MC, Berger W, Carrel T, Steinmann B, Mátyás G. Quantitative sequence analysis of FBN1 premature termination codons provides evidence for incomplete NMD in leukocytes. Hum Mutat. 2009;30:1355-64.

11. Schrijver I, Liu W, Brenn T, Furthmayr H, Francke U. Cysteine Susstitutions in epidermal growth factor-like domains of fibrillin-1: distinct effects on biochemical and clinical phenotypes. Am J Hum Genet. 1999;65:1007-20.

12. Rommel K, Karck M, Haverich A, von Kodolitsch Y, Rybczynski M, Müller G, Singh KK, Schmidtke J, Arslan-Kirchner M. Identification of 29 novel and nine recurrent fibrillin-1 (FBN1) mutations and genotype-phenotype correlations in 76 patients with Marfan syndrome. Hum Mutat. 2005;26:529-39.

13. Arbustini E, Grasso M, Ansaldi S, Malattia C, Pilotto A, Porcu E, Disabella E, Marziliano N, et al. Idetnification of sixty-two novel and twelve known FBN1 mutations in eighty –one unrelated probands with Marfan síndrome and ohter fibrillinopathies. Hum Mutat;26:494.

14. Lerner-Ellis JP, Aldubayan SH, Hernandez AL, Kelly MA, Stuenkel AJ, Walsh J, Joshi VA. The spectrum of FBN1, TGFβR1, TGFβR2 and ACTA2 variants in 594 individuals with suspected Marfan Syndrome, Loeys–Dietz Syndrome or Thoracic Aortic Aneurysms and Dissections (TAAD). Mol Genet Metab. 2014;112:171-6.

15. Milewicz DM, Grossfield J, Cao SN, Kielty C, Covitz W, Jewett T. A mutation in FBN1 disrupts profibrillin processing and results in isolated skeletal features of the Marfan syndrome. J Clin Invest. 1995;95:2373-8.

16. Körkkö J, Kaitila I, Lönngvist L, Peltonen L, Ala-Kokko L. Sensitivity of conformation sesitive gel electrophoresis in detecting mutations in MArfan síndrome and related conditions. J Med Gent. 2002;39:34-41.

17. Stheneur C, Collod-Béroud G, Faivre L, Buyck JF, Gouya L, Le Parc JM, Moura B, Muti C, Grandchamp B, Sultan G, Claustres M, Aegerter P, Chevallier B, Jondeau G, Boileau C. Identification of the minimal combination of clinical features in probands for efficient mutation detection in the FBN1 gene. Eur J Hum Genet. 2009;17:1121-8.
